# *CD95* gene deletion may reduce clonogenic growth and invasiveness of human glioblastoma cells in a CD95 ligand-independent manner

**DOI:** 10.1038/s41420-022-01133-y

**Published:** 2022-07-29

**Authors:** Clara Quijano-Rubio, Manuela Silginer, Michael Weller

**Affiliations:** 1 Laboratory of Molecular Neuro-Oncology, Department of Neurology, University Hospital Zurich, Zurich, Switzerland; 2 University of Zurich, Zurich, Switzerland

**Keywords:** CNS cancer, Cancer stem cells, Cell signalling, Cell invasion, Cell growth

## Abstract

CD95 (Fas/APO-1) is a multifunctional cell surface receptor with antithetic roles. First described to mediate cell death, interactions of CD95 with its natural ligand, CD95L, have also been described to induce tumor-promoting signaling leading to proliferation, invasion and stem cell maintenance, mainly in cancer cells that are resistant to CD95-mediated apoptosis. While activation of CD95-mediated apoptosis in cancer cells may not be clinically practicable due to toxicity, inhibition of tumor-promoting CD95 signaling holds therapeutic potential. In the present study, we characterized CD95 and CD95L expression in human glioma-initiating cells (GIC), a glioblastoma cell population with stem cell features, and investigated the consequences of CRISPR-Cas9-mediated *CD95* or *CD95L* gene deletion. In vitro, GIC expressed *CD95* but not *CD95L* and were sensitive to CD95-mediated apoptosis. Upon genetic deletion of *CD95*, GIC acquired resistance to CD95L-induced apoptosis but exhibited inferior clonogenic growth, sphere-forming capacity, and invasiveness compared with control cells, suggesting the existence of CD95L-independent constitutive CD95 signaling with tumor-promoting properties in GIC. In vivo, GIC expressed CD95 and a non-canonical form of CD95L lacking the CD95-binding region. *CD95* genetic deletion did not prolong survival in immunocompromised GIC-bearing mice. Altogether, these data indicate that canonical CD95L may not be expressed in human GIC and suggest the existence of a CD95L-independent CD95-signaling pathway that maintains some malignancy traits of GIC. The lack of altered survival of tumor-bearing mice after genetic deletion of *CD95* suggests that CD95 signaling is not essential to maintain the growth of human GIC xenografted into the brains of nude mice. The ligand-independent tumor-promoting role of constitutive CD95 in our GIC models in vitro highlights the complexity and challenges associated with targeting CD95 with therapeutic intent.

## Introduction

Glioblastoma is the most common and aggressive type of malignant primary central nervous system tumor in adults [1]. Despite the implementation of the current standard of care, which includes maximal surgical resection, radiotherapy, and alkylating agent chemotherapy, the prognosis remains poor and virtually all patients experience tumor recurrence and eventually die from the disease [2, 3]. Glioblastomas contain heterogeneous tumor cell populations with distinct gene expression profiles [4, 5]. This heterogeneity may contribute to resistance to therapy and recurrence, as it permits clonal selection that may in part be driven by glioma-initiating cells (GIC). GIC represent a tumor cell population possessing neuroglial stem cell properties and are characterized by self-renewal capacity, indefinite proliferation, and multipotency [6]. Hence, the investigation and development of therapeutic strategies targeting GIC are of relevance in the context of glioblastoma.

CD95 (Fas/APO-1) is a pleiotropic molecule with antithetic functions. Traditionally, CD95 has been recognized as a prototypic death receptor but additional evidence has positioned cognate interactions between CD95 and CD95 ligand (CD95L) as tumor-promoting. Specifically, non-apoptotic CD95L–CD95 signaling has been linked to cancer cell proliferation, invasiveness, and stemness [7, 8]. The factors determining whether CD95 signaling results in apoptotic or tumor-promoting signaling remain to be fully defined. Cellular architecture [9], the operation of initiator caspases [10–12], CD95 phosphorylation status [13], CD95L processing [14–19], and the intensity of the CD95L stimulus [20, 21] have been proposed as regulatory mechanisms.

Apoptotic signaling via CD95 has been extensively studied in glioblastoma models decades ago [22–26]. However, more recent studies have focused on tumor-promoting CD95 signaling [7, 27–30] and provided the framework for the neutralization of CD95L, resulting in a signal of clinical activity in combination with radiotherapy in recurrent disease [31].

The role of CD95 signaling in studies reporting tumor-promoting or tumor-suppressing CD95 signaling in glioblastoma was inferred upon pharmacologic targeting, RNAi-mediated gene downregulation, ectopic CD95 or CD95L expression, or the comparative analysis of tumor cell populations segregated based on CD95 expression levels. However, the role of CD95 signaling in glioblastoma has not been explored in an experimental approach that specifically and entirely abrogates the *CD95L* or *CD95* gene. Here we explored the role of constitutive CD95 signaling in human glioblastoma models with a specific focus on invasiveness, stemness-associated features, and tumorigenicity.

## Results

### CD95 and CD95L expression in glioblastoma in vivo and in vitro

The analysis of overall survival in glioma patients from two different cohorts of The Cancer Genome Atlas (TCGA) database demonstrated inferior survival by the trend in patients with higher *CD95* mRNA expression levels among isocitrate dehydrogenase (IDH)-mutant gliomas, but not in glioblastoma, IDH wildtype, assessed by setting either median *CD95* mRNA expression or the highest association with survival as a cut-off (Fig. S1A, C). Conversely, patients with high *CD95L* mRNA expression had better overall survival by trend in IDH-mutant gliomas (Fig. S1B, D).

**Fig. 1:**
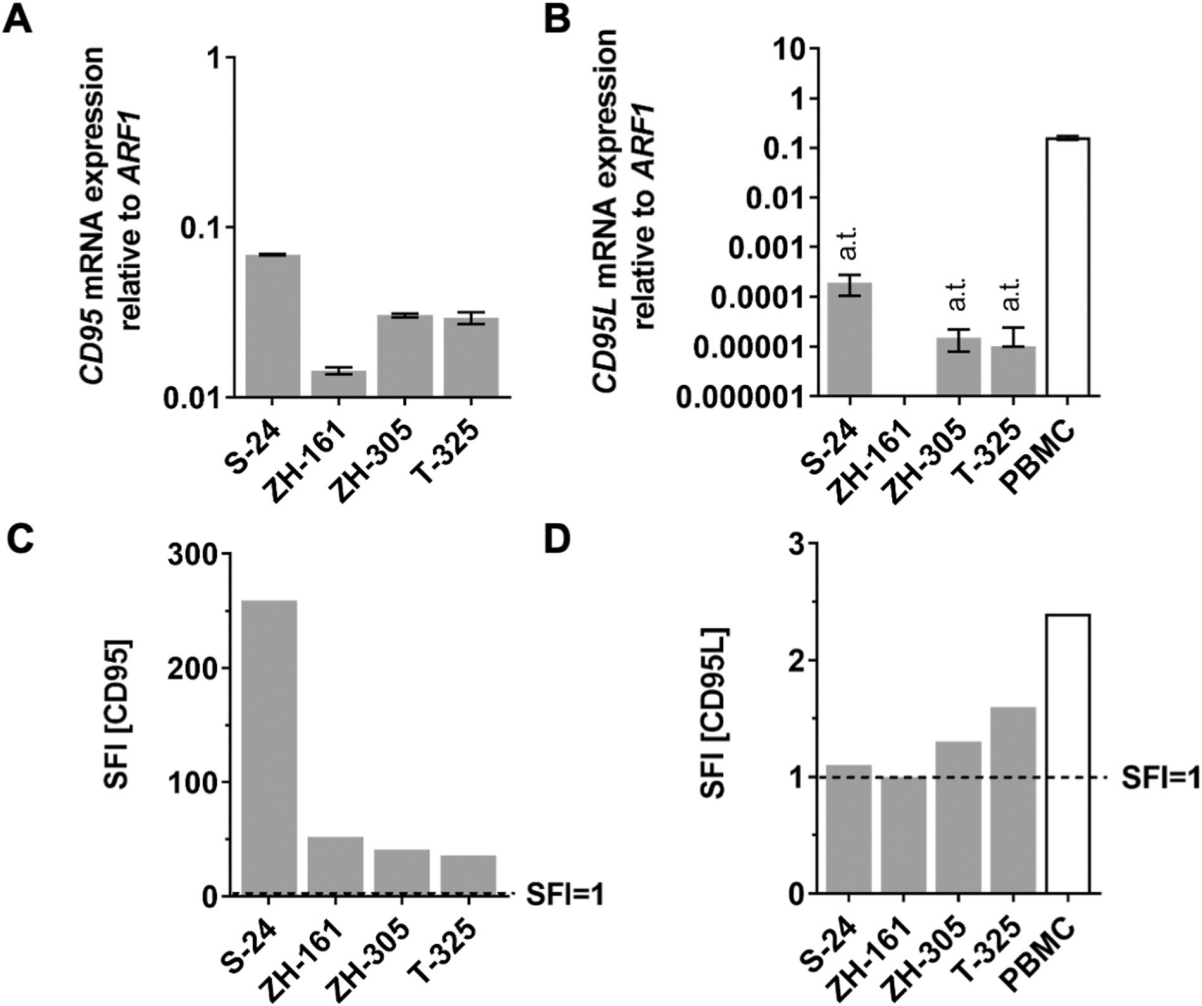
CD95 and CD95L expression in human GIC in vitro. **A**, **B** S-24, ZH-161, ZH-305, or T-325 human GIC were assessed for expression of *CD95* or *CD95L* mRNA by RT-qPCR using primers targeting all known protein-coding *CD95* or *CD95L* transcript variants and *ARF1* as an internal control. Phorbol myristate acetate (10 ng/ml) and ionomycin (500 ng/ml)-activated peripheral blood mononuclear cells (PBMC) served as a positive control for *CD95L* expression detection. RT-qPCR data are expressed as mean and SD. a.t., above the threshold, indicates C_T_ values above the reliability threshold of 32. **C**, **D** Cell surface protein levels were assessed by flow cytometry. Specific fluorescence indexes (SFI) were calculated by dividing the median fluorescence intensities of the experimental antibody and the isotype control.

CD95 expression was detected in human GIC lines (S-24, ZH-161, ZH-305, T-325) on mRNA (Fig. 1A) and cell surface protein levels (Fig. 1C, Fig. S2A). CD95L expression was examined in a broad panel of human GIC and long-term glioblastoma cell lines. Neither mRNA nor cell surface protein expression was detected in any GIC (Fig. 1B, D, Fig. S2B) or other human long-term glioblastoma cell lines (Fig. S2C, Note S1).

**Fig. 2:**
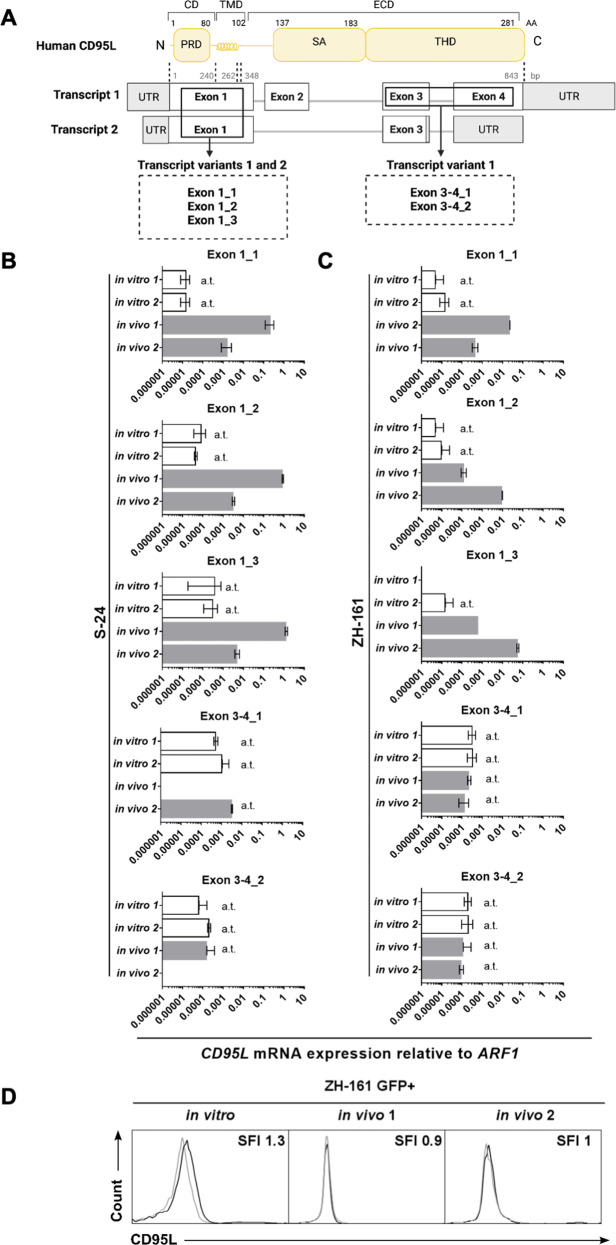
*CD95L* expression in human GIC in vivo. **A** Schematic representation of the two *CD95L* transcript variants and transcript sequence correspondence with CD95L protein domains (created with BioRender.com). The binding sites of five primer pairs and the transcript variants they are predicted to amplify are schematically indicated. **B**, **C** S-24 and ZH-161 cells were assessed for expression of human *CD95L* mRNA in vitro or in tumor-bearing mouse brain hemispheres by RT-qPCR using three human-specific primers targeting the first exon of human *CD95L* (exon 1_1, exon 1_2, and exon 1_3) or two human-specific primers spanning the third and the fourth exon of human *CD95L* (exon 3-4_1 and exon 3-4_2). Two in vitro cultures (in vitro 1 and 2) and two tumor-bearing mouse brains (in vivo 1 and 2) per model were studied. *ARF1* was used as an internal control. **D** GFP-labeled ZH-161 cells were intracranially implanted in nude mice, isolated upon neurologic symptom onset, and analyzed by flow cytometry. Flow cytometry histograms depicting cell surface CD95L levels in GFP + ZH-161 cells are shown. Data are expressed as mean and SD. a.t., C_T_ values above reliability threshold (C_T_ > 32). CD death domain, TMD transmembrane domain, ECD extracellular domain, PRD proline-rich domain, SA self-assembly domain, THD TNF homology domain.

Two *CD95L* transcript variants encoding two distinct protein isoforms have been described: a longer canonical transcript variant, which encodes the full-length transmembrane CD95L protein, and a shorter transcript variant, which encodes a protein lacking part of the extracellular domain [32, 33]. To confirm that none of the *CD95L* variants were expressed in human GIC, a set of five primer pairs targeting different *CD95L* exons was designed: three individual primer pairs targeting different regions within the first *CD95L* exon, which is present in both transcript variants, and two individual primer pairs targeting the region spanning the third and fourth *CD95L* exons, which is only present in the canonical *CD95L* transcript variant (Fig. 2A). None of the primer pairs led to *CD95L* mRNA amplification in two selected human GIC in vitro (Fig. 2B, C; upper bars in each graph), indicating that neither S-24 nor ZH-161 cells express either of the CD95L transcript variants in vitro. In Fig. 1B and in Fig. S2E, *CD95L* transcript levels quantified using a primer pair targeting exon 1 (exon 1_1) and a primer pair targeting exons 3-4 (exon 3-4_1), respectively, are depicted, which extends the latter interpretation on the absence of expression of all *CD95L* transcript variants to all selected GIC. *CD95L* transcript expression in PBMC was observed with both primer pairs, confirming their technical reliability.

### *CD95L* is upregulated in human GIC in vivo

In contrast to the absence of human CD95L expression in vitro, the analysis of *CD95L* transcript levels in the tumors of glioma-bearing mice revealed an upregulation of *CD95L* in vivo in S-24 and ZH-161 xenografts (Fig. 2B, C). However, *CD95L* transcript amplification was detected with human-specific primers targeting the first exon only, but not with human-specific primers spanning the third and fourth exon of the *CD95L* transcript sequence, indicating that the expression of *CD95L* in vivo corresponds to a non-canonical *CD95L* transcript variant lacking most of the extracellular domain. Accordingly, full-length CD95L was not detected by flow cytometry on the surface of GIC isolated from end-stage tumor-bearing mice (Fig. 2D).

### CD95 and CD95L knockout in human GIC

**Fig. 3:**
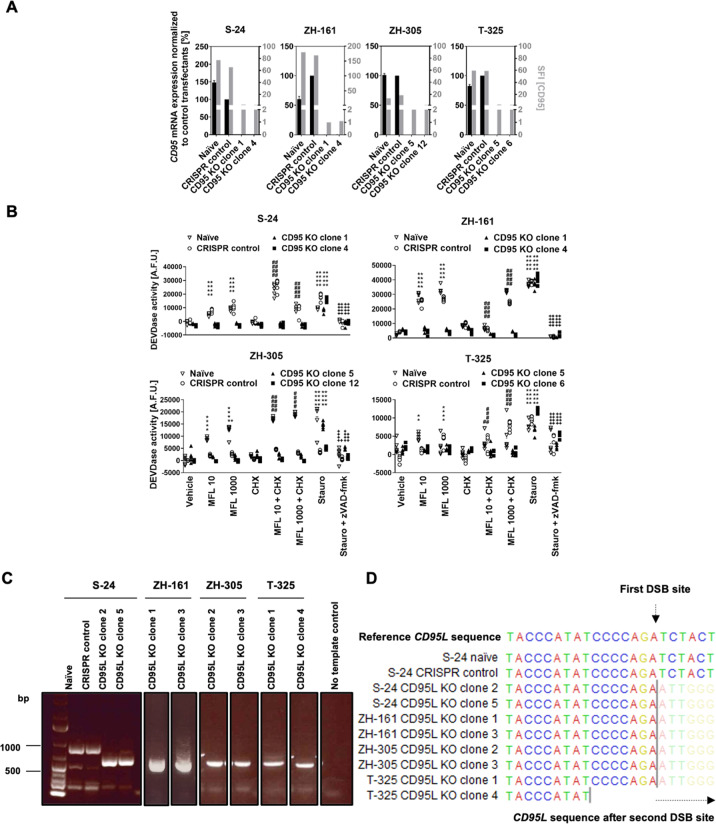
CD95 and CD95L CRISPR knockout (KO) in selected human GIC. The *CD95* or *CD95L* genes were depleted via CRISPR-Cas9 in S-24, ZH-161, ZH-305, or T-325 cells. **A** CD95 clonal knockout sublines were assessed for expression of the *CD95* transcript spanning the two predicted double-strand DNA break (DSB) sites by RT-qPCR using *ARF1* as internal control and of cell surface protein by flow cytometry. RT-qPCR data are expressed as mean and SD. Specific fluorescence indexes (SFI) were calculated by dividing the median fluorescence intensities of the experimental antibody and the isotype control. **B** CD95 KO was functionally validated by assessing the sensitivity of naïve, CRISPR control or CD95 KO S-24, ZH-161, ZH-305, or T-325 cells to exogenous CD95L-mediated apoptosis. Naïve, CRISPR control or CD95 KO S-24, ZH-161, ZH-305, or T-325 cells were stimulated with 10 or 1000 ng/ml exogenous CD95L (Mega-Fas-Ligand, MFL) in the absence or presence of cycloheximide (CHX) (10 µg/ml) for 6 h and assessed for caspase 3/7-like Ac-DEVD-amc-cleaving activity (DEVDase activity). As a positive control, cells were treated with 1 μM staurosporine (stauro). As a negative control, cells were treated with the pan-caspase inhibitor zVAD-fmk (10 μM) in combination with staurosporine. A representative experiment is shown for each cell line. Data of six technical replicates (triangles) are shown. Statistical significances were calculated by two-way ANOVA followed by Bonferroni’s post hoc test (*p < 0.05, **p < 0.01, ****p < 0.0001 versus vehicle; ^####^*p* < 0.0001 versus CHX; ^+^*p* < 0.05, ^+++^*p* < 0.001, ^++++^*p* < 0.0001 versus stauro). **C**, **D** CD95L clonal knockout sublines were selected based on the absence of the *CD95L* genomic DNA sequence spanning the two predicted DSB sites. The *CD95L* gene fragment spanning the two predicted DSB sites was amplified by PCR using primers flanking the single guide RNA (sgRNA) target sequences and amplicon size was verified by agarose gel electrophoresis (**C**). Amplicon sequence was elucidated by Sanger sequencing with primers targeting the upstream sequence of the first sgRNA target sequence (**D**). Naïve refers to non-transfected cells, while CRISPR control refers to cells transfected with non-targeting sgRNA in pSpCas9(BB)-2A-GFP plasmids. A.F.U., arbitrary fluorescence units.

Next, to explore the role of CD95 and CD95L in human GIC, CD95 or CD95L were depleted in S-24, ZH-161, ZH-305, or T-325 cells by means of CRISPR-Cas9 (Fig. S3, Note S3). CD95 knockout clonal sublines were selected based on the absence of the transcript region spanning two predicted double-strand DNA break sites, defined by the two target sequences utilized, and of cell surface protein (Figs. 3A, S4). The CD95 knockout was functionally confirmed by demonstrating abrogation of DEVD-amc peptide-cleaving activity, which characterizes the activity of the apoptotic effector caspase 3 that mediates canonical CD95 signaling, in all selected CD95 knockout clonal sublines upon stimulation with exogenous CD95L alone and in combination with cycloheximide, which sensitizes glioma cells to apoptosis induction [22] (Fig. 3B). Because of the absence of CD95L expression in human GIC in vitro, CD95L knockout clonal sublines were identified and selected based on the absence of the *CD95L* genomic DNA spanning the two predicted double-strand DNA break sites (Fig. 3C, D).

### Characterization of the CD95 knockout phenotype in human GIC in vitro

**Fig. 4:**
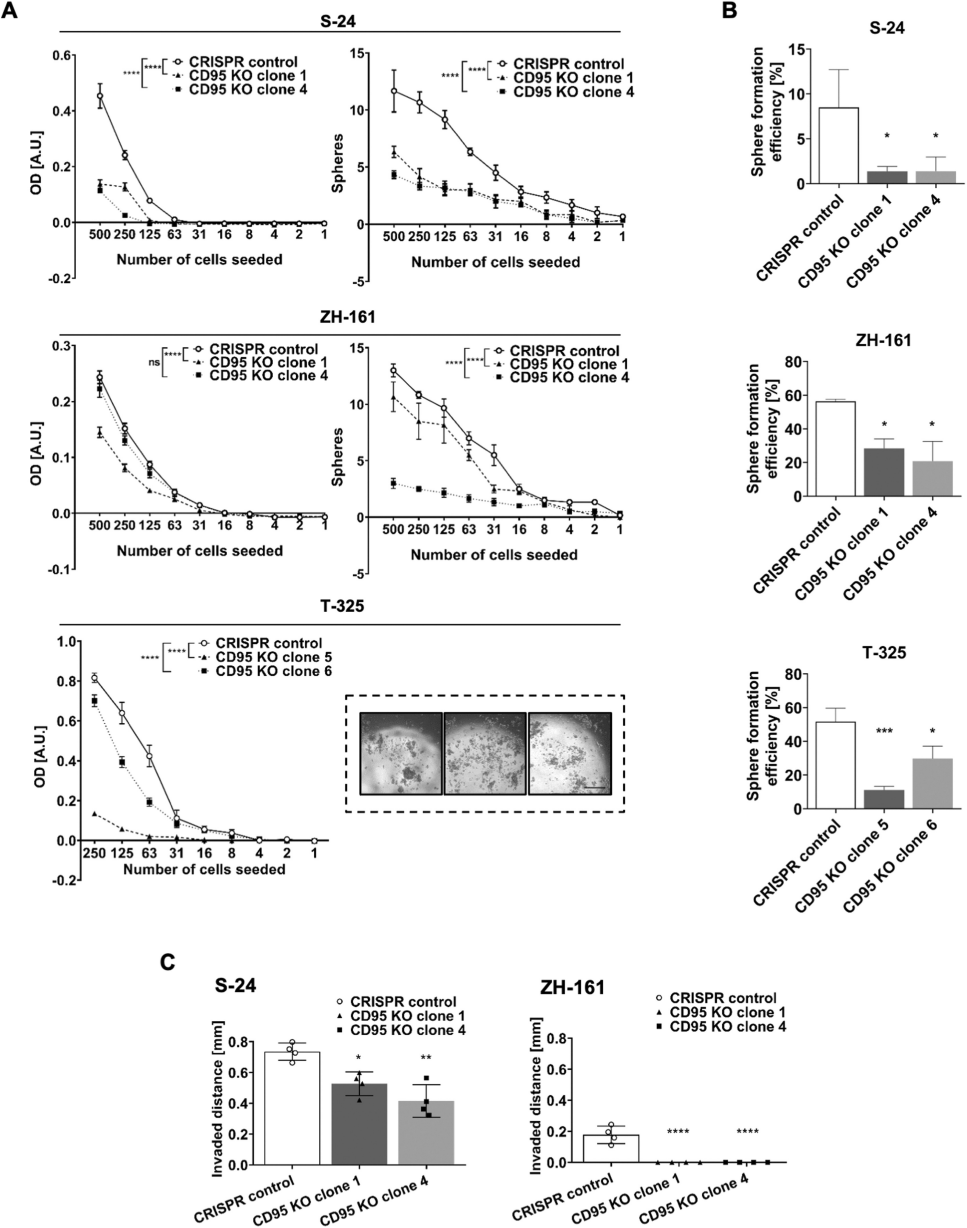
Effect of CD95 CRISPR knockout (KO) on human GIC growth, spherogenicity and invasion in vitro. **A** The clonogenic growth and sphere formation capacity of S-24, ZH-161, and T-325 cells were evaluated by their end-point metabolic activity as measured by MTT-based quantification and by sphere counting in limiting dilution assays. Data in **A** are expressed as mean and SEM of representative experiments. Statistical significances were determined by means of a two-way ANOVA test followed by Bonferroni’s post hoc test (main column effect). **B** Sphere formation efficiency upon single-cell seeding was calculated as the number of spheres counted/number of seeded cells. Data in **B** are expressed as the mean and SD of three experiments. **C** The invasion capacity of CRISPR control or CD95 KO S-24 or ZH-161 cells was assessed by means of collagen invasion assays. Invaded distances from the spheroid center were quantified. Data in **C** are expressed as the mean and SD of a representative experiment. Statistical significances in **B**, **C** were determined by a one-way ANOVA test followed by Bonferroni’s post hoc test. Data in **A**–**C** were reproduced in three independent experiments. *p < 0.05, **p < 0.01, ***p < 0.001, ****p < 0.0001, ns not significant versus CRISPR control cells; A.U., arbitrary units; Inset scale bar = 500 μm.

The expression data summarized above suggested that CD95L knockout cells should have no phenotype since CD95L is not expressed (Note S4) and that therefore any phenotype in CD95 knockout cells must be CD95L-independent. CD95 depletion did not affect doubling times in either GIC model (Fig. S5), but resulted in decreased clonogenic growth and sphere-forming capacity in limiting dilution assays in S-24 and ZH-161 (Fig. 4A), although not in ZH-305 cells (data not shown). Sphere-forming capacity could not be assessed in T-325 cells since these did not form quantifiable spheres with defined borders (Fig. 4A; inset, S6A). To circumvent the limitation imposed by the growth pattern of T-325 cells, as well as to ensure the clonal origin of the formed spheres, GIC sphere-forming capacity was additionally inferred from their sphere formation efficiency, defined as the percentage of cells capable of leading to sphere formation upon single cell seeding. By these means, reduced sphere formation efficiency was revealed in T-325 cells, as well as in S-24 and ZH-161 cells, although not in ZH-305 cells upon CD95 knockout (Fig. 4B, data not shown), in agreement with the limiting dilution assay results.

Spheroid collagen invasion assays revealed that CD95 depletion was associated with a less invasive phenotype in S-24 and complete inhibition of invasion in ZH-161 cells, which possess a low invasive potential in naïve conditions already (Fig. 4C, Fig. S6B). The invasiveness of ZH-305 and T-325 cells upon CD95 knockout could not be evaluated since collagen invasion was not observed in any of the sublines (Fig. S6C).

Inactivation of glycogen synthase kinase (GSK)3β through serine 9 phosphorylation following CD95/CD95L interactions has been reported to mediate invasion in long-term glioma cells [27]. Here, differences in GSK3β S9 phosphorylation between CD95 KO and control S-24 GIC were not revealed, neither constitutively nor upon stimulation with Mega-Fas-Ligand (data not shown).

Alterations in the expression levels of diverse stemness (*CD133*, *CD44*, *SOX2*, *MUSASHI1*, *OCT4*) and differentiation (*OLIG2*, *NF1*, *TUBB3*, *GFAP*) markers [34, 35] were not observed upon CD95 knockout in either GIC model (S-24, ZH-305, T-325) (Fig. S7).

### Neither CD95 nor CD95L knockout affects survival in xenograft glioma murine models

**Fig. 5:**
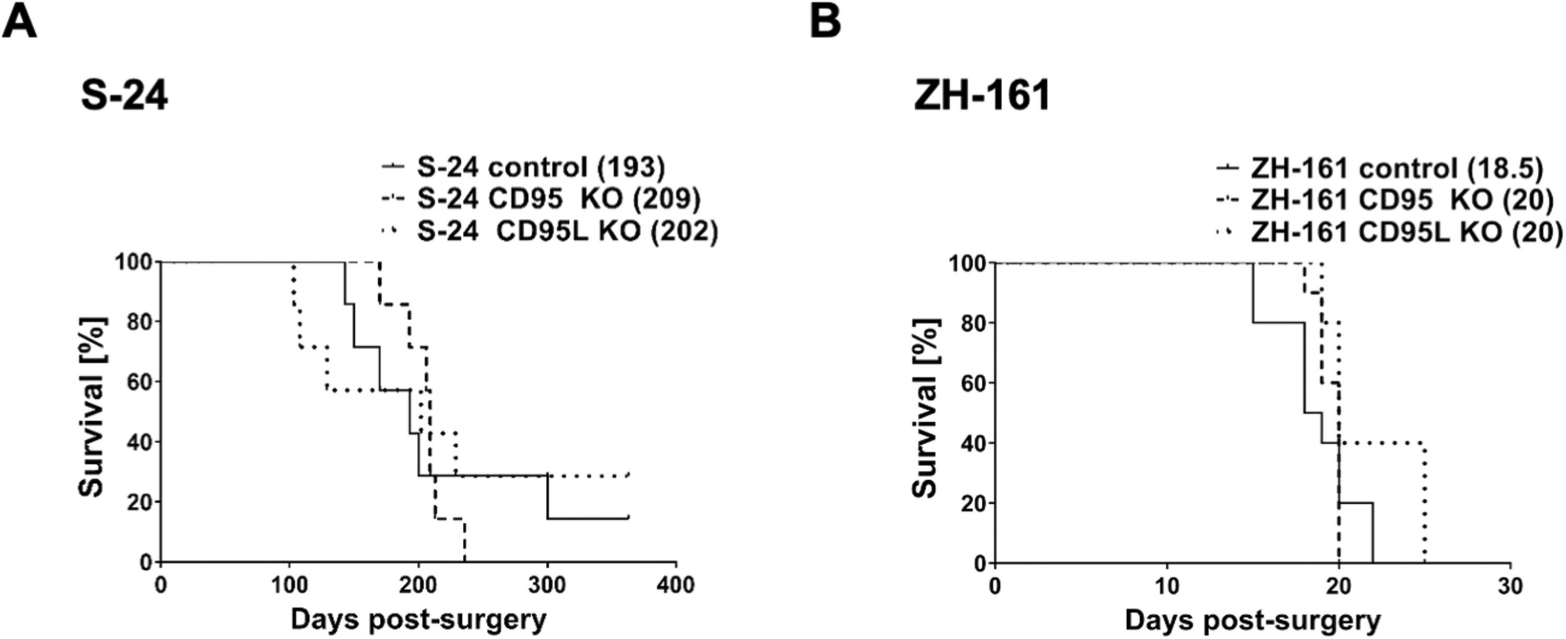
Effect of CD95 or CD95L knockout (KO) in human GIC xenograft models. Crl:CD1-*Foxn1*^*nu*^ or Rj:NMRI-*Foxn1*^*nu*^ nude mice were intracranially implanted with control, CD95 KO or CD95L KO S-24 (**A**) or ZH-161 (**B**) cells. The control group includes mice implanted with naïve or CRISPR control cells, the CD95 KO group includes mice implanted with two different CD95 KO clones and the CD95L KO group includes mice implanted with two different CD95L KO clones. End-stage survival of *n* = 7 mice per group was recorded. Median survival in days is depicted, in brackets, next to the respective experimental group. The survival of control and CD95 KO or CD95L KO glioma-bearing mice was compared by means of a log-rank test. Statistical significance was not revealed.

To study the functional outcome of gene deletion in vivo, S-24, or ZH-161 cells were orthotopically implanted into the brains of immunocompromised *Foxn1*^*nu*^ mice. The median survivals of control, CD95 knockout, and CD95L knockout tumor-bearing mice did not differ significantly, being 193, 209, and 202 days, respectively, in the S-24 model and 18.5, 20, and 20 days in the ZH-161 model (Fig. 5A, B). Because of the low tumorigenicity of ZH-305 and T-325 cells, the effect of gene deletion in vivo could not be studied in these models (Fig. S9).

## Discussion

Although traditionally regarded as a prototypic death receptor, CD95 has been also described to mediate non-apoptotic pleiotropic effects with tumor-promoting potential. This notion implies the therapeutic potential for the blockade of CD95 signaling. Accordingly, CD95 and CD95L have been reported as negative prognostic markers in different studies on various tumor entities, including glioblastoma [7, 36]. However, reports on CD95 or CD95L expression as positive prognostic factors in cancers including lung cancer, leukemia, and lymphoma have also been published [37–39]. We analyzed the overall survival in two TCGA glioblastoma cohort patients divided based on CD95 and CD95L expression levels and did not identify a major prognostic role of either molecule (Fig. S1).

Through extensive gene expression and protein analyses, we demonstrated that GIC express CD95, but not canonical CD95L. In apparent contrast to our observations, CD95L expression has repeatedly been reported in glioblastoma specimens and cell lines over the last years [7, 27, 29, 31, 40, 41]. While the specificity of the antibodies used in the early studies has been questioned [42], CD95L expression in fresh specimens may be attributed to stromal cells or to an upregulation of CD95L in vivo. We detected the upregulation in GIC in vivo only of a non-canonical transcript variant encoding a CD95L isoform lacking most of the extracellular domain (Figs. 1 and 2).

To investigate the role of constitutive CD95 cancer cell-intrinsic signaling in glioblastoma, we deleted *CD95* in four human GIC models which are thought to resemble the original tumors better than long-term cell line models [43, 44], by means of CRISPR-Cas9. In three out of the four GIC models studied, we observed a reduction in clonogenic growth and sphere-forming capacity under low cell density conditions upon CD95 knockout (Fig. 4A, B). Observations similarly suggesting that CD95 modulates spherogenicity have been made upon pharmacologic modulation of CD95 and CD95L using anti-APO-1, LzCD95L, or APG101, or upon sorting of glioblastoma cancer cell populations based on CD95 expression levels [7, 28]. Additionally, CD95 knockout reduced or abrogated invasion in all GIC exhibiting constitutive invasive capacity into collagen matrixes in vitro (Fig. 4C). These data are phenotypically consistent with increased Yes/phosphatidylinositol 3‑kinase (PI3K)/GSK3β/matrix metalloproteinase (MMP)-mediated migration upon pharmacologic CD95 stimulation in long-term glioma cell lines [27] and upon inhibition of exogenous CD95L-induced invasion by pharmacological CD95L neutralization [29, 30]. Nonetheless, we did not observe an association between GSK3β activity and CD95-mediated invasiveness in GIC (data not shown). Invasion is a feature associated with cancer cell stemness [45]. However, we did not observe alterations in stemness and differentiation gene signatures upon CD95 knockout in our models (Fig. S7). These data contrast with reports correlating CD95 expression with cancer stem cell gene signatures, although the latter derive from analyses on tumor sub-populations segregated based on CD95 expression and may therefore entail additional population differences [7]. All in all, our results indicate that constitutive CD95 signaling in human GIC may be tumor-promoting. Furthermore, although it has been argued that tumor-promoting CD95 signaling is exclusive to cells resistant to CD95-mediated apoptosis [10, 27, 46, 47], we demonstrate that CD95 mediates clonogenic growth, sphere formation, and invasion in human GIC that are intrinsically sensitive to CD95L-induced apoptosis (Fig. 3). This existence of tumor-promoting CD95 activities in CD95-mediated apoptosis-sensitive cells has been also suggested in other tumor entities [48].

Importantly, our data further suggest that constitutive CD95-mediated clonogenic growth, sphere-forming capacity, and invasion in human GIC may be independent of CD95L, since none of the GIC investigated in this study expressed CD95L in vitro. Stimulation of CD95-expressing GIC with sublethal concentrations of exogenous CD95L did not result in increased cell growth either, supporting the CD95L independency of the observations reported here (Fig. S8). Overexpressing CD95 in GIC did not enhance clonogenic growth, suggesting that CD95L-independent tumor-promoting CD95 signaling may sustain constitutive cancer cell growth, but does not do so in a simple linear dose-dependent manner (Fig. S8). Comparable observations were made in the context of CD95L-dependent CD95 signaling in breast and renal cancer where CD95 overexpression did not increase cancer cell proliferation [48].

The hypothesis of a CD95L-independent tumor-promoting CD95 signaling may entail the existence of alternative interaction partners of CD95. Crosstalk between CD95 and tumor necrosis factor receptor 1 (TNF-R1) [49], CD40 [50], MET [51], and epidermal growth factor receptor (EGFR) [52] has been reported. Further, since CD95 and integrins share intracellular partners, it has been hypothesized that CD95 may be cross-activated by integrins [53]. Additionally, CD95 is presented as organized oligomeric structures prior to CD95L binding [54] and apoptosis inhibition upon N-terminal oligomerization of identical and distinct soluble CD95 isoforms have been reported, too [55]. Whether or not homotypic or heterotypic CD95 interactions in the absence of CD95L may mediate constitutive tumor-promoting signal transduction remains open.

CD95L-independent interactions between CD95 and the Kip1 ubiquitination-promoting complex protein 2 (KPC2) have been recently reported and associated with NF-κB suppression [56]. NF-κB has been suggested to regulate *CD44* [57], *SOX2* [58], *OCT4* [58, 59], and *OILG2* [60] expression in association with cancer cell invasiveness or stemness in different cancer models. Nevertheless, we did not observe differences in the expression of any of the above-mentioned genes upon CD95 gene deletions in our models (Fig. S7).

CD95L backward signaling has been described in T cells [61], but it remains unclear whether this phenomenon is relevant under physiological or pathological conditions. We did not observe a phenotype of *CD95L* gene deletion in vitro or in vivo (Note S4, Fig. 5). Despite detriment in cell growth and invasive potential upon CD95 depletion in S-24 and ZH-161 cells in vitro, the median survival of mice orthotopically implanted with CD95 knockout S-24 or ZH-161 GIC did not differ from control mice (Fig. 5).

All in all, our data indicate that CD95 positively regulates cell growth, spherogenicity, and invasion in human GIC in vitro in a CD95L-independent manner, suggesting that direct CD95 blockade may represent a superior approach to CD95L neutralization if CD95 signaling in tumor cells is the therapeutic target. Depletion of cell-intrinsic CD95 signaling alone is nevertheless insufficient to provide a survival benefit in GIC xenograft models in immune-incompetent models. Therefore, investigating whether the disruption of cell-intrinsic CD95 signaling augments survival in models with an intact adaptive immune response is warranted to comprehensively evaluate the therapeutic potential of constitutive CD95 signaling disruption.

## Material and methods

### Reagents

Cycloheximide, staurosporine, and carbobenzoxy-valyl-alanyl-aspartyl-[O-methyl]-fluoromethylketone (zVAD-FMK) were purchased from Santa Cruz Biotechnology (Dallas, TX), VWR (Radnor, PA) and Bachem (Bubendorf, Switzerland). Mega-Fas-Ligand was kindly provided by TopoTarget S.A. (Lausanne, Switzerland).

### Cell lines

Human GIC (S-24, T-269, T-325, ZH-161, and ZH-305) were established from freshly resected tumors [62]. The human long-term glioma cell lines LN-18, LN-428, D247MG, LN-319, A172, LN-308, and LN-229 [63] were kindly provided by N. de Tribolet (Lausanne, Switzerland) and T98G cells were obtained from the American Type Culture Collection (ATCC) (Rockville, MD). GIC were cultured as neurospheres in Neurobasal medium (NB) supplemented with 2 mM l-glutamine, 20 μg/ml B-27 supplement (Gibco, Waltham, MA), 20 ng/ml fibroblast growth factor (FGF) 2, and 20 ng/ml epidermal growth factor (EGF) (Peprotech, Rocky Hill, PA). Long-term glioma cell lines were grown as adherent monolayers in Dulbecco´s modified Eagle´s medium (DMEM) supplemented with 10% fetal calf serum (FCS) and 2 mM l-glutamine (Gibco). Cells were regularly tested for mycoplasma contamination by means of MycoAlert^TM^ PLUS Mycoplasma Detection (Lonza, Basel, Switzerland).

### *CD95* and *CD95L* gene deletion

*CD95* or *CD95L* genes were knocked out in S-24, ZH-161, ZH-305, and T-325 human GIC by CRISPR-Cas9 technology [64]. Two sgRNA complementary to two distinct coding sequences present in all target gene transcript variants of human *CD95* or *CD95L* were designed using the online CRISPR design tool http://tools.genome-engineering.org with minimal predicted off-target binding. The sgRNA sequences were designed as: 5’-GATTGCTCAACAACCATGCT-3’, sense and 5’-GGAGTTGATGTCAGTCACTT-3’, antisense (*CD95* deletion); 5’-GCTGTCCACCCAGTAGATCT-3’, antisense and 5’-CTGGTTGCCTTGGTAGGATT-3’, sense (*CD95L* deletion). GIC were transfected with sgRNA-encoding pSpCas9(BB)-2A GFP (PX458) plasmids (#48139, Addgene, Watertown, MA) by electroporation using a Neon transfection system (Thermo Fisher Scientific). Briefly, three million GICs were transfected with 18 μg DNA, consisting of a 1:1 mix of the two plasmids containing each sgRNA sequence per target gene. Voltage, width, and pulse number were 1600 V, 10 ms, and 3, respectively. Transfected (GFP+) cells were selected by fluorescence-activated cell sorting (FACS) 3–5 days post-transfection and seeded as single cells for the generation of *CD95* and *CD95L* KO clonal cell populations. Knockout verification was performed by RT-qPCR using primers spanning the predicted Cas9-mediated double-strand DNA break sites, flow cytometry, or Sanger sequencing. For the generation of CRISPR control cells, the following non-targeting sgRNA were used: 5’-ACGGAGGCTAAGCGTCGCAA-3’ and 5’-ATCGTTTCCGCTTAACGGCG-3’. CRISPR control cells were used as bulk populations.

### RT-qPCR

Total mRNA was isolated using the NucleoSpin^®^RNA II kit (Macherey-Nagel, Dueren, Germany). Fifteen nanograms of cDNA, generated using a High Capacity cDNA Reverse Transcription kit (Thermo Fisher Scientific), were amplified with the PowerUp SYBR Green Master Mix in a QuantStudio 6 Real-Time thermocycler (Thermo Fisher Scientific) applying the following conditions: 50 °C/2 min, 95 °C/2 min and 40 cycles at 95 °C/15 s and 60 °C/1 min. Relative transcript expression quantification was computed using the primer efficiency-weighted comparative C_T_ (∆C_T_) method. Specific transcript expression was normalized to ADP-ribosylation factor 1 (*ARF1*) [65, 66]. A list of the primers used is provided in Table S1.

### Flow cytometry

Cell dissociation with Accutase (Thermo Fisher Scientific) was followed by incubation with the following antibodies at 4 °C for 30 min: APC mouse anti-human CD95 clone DX2 (# 56197 1:50), PE mouse anti-human CD95L clone NOK-1 (#306406 1:100) or matching isotype controls, all from BD Biosciences. Cells were incubated with Zombie Aqua (BioLegend) in parallel for live/dead staining. Data acquisition and analysis were performed with a BD FACSVerse flow cytometer (BD Biosciences) and FlowJo (Tree Starm, Stanford, CA). Unless specified otherwise, only specific fluorescence indexes (SFI) higher than 1.5 were considered potentially indicative of protein expression.

### Cell growth assessment in limiting dilution assays

A total of 500 to 1 cells/well were seeded by means of limiting dilution in 96-well plates and incubated for >10 days. End-point GIC metabolic activity was assessed based on thiazolyl blue tetrazolium bromide (MTT) reduction.

### Spherogenicity assays

GIC was seeded in limiting dilution assays as described above or as single cells. After an incubation period of 10 days or more, the number of spheres in each well, defined as clusters of at least five cells, was quantified under light microscopy. In limiting dilution-based spherogenicity assays, total numbers of spheres per well were quantified. In single-cell seeding-based spherogenicity assays, the number of spheres formed as a percentage of cells seeded was reported.

### Collagen invasion assays

Four similar size spheroids were obtained upon cell seeding in cell repellent 96-well round-bottomed plates. Spheroids were thereafter embedded into 2.7 mg/ml type I bovine collagen (Advanced BioMatrix, Carlsbad, CA). Once polymerized, the cell collagen matrix was overlayed with a 10% FCS-containing culture medium or NIH 3T3 cell supernatant. The invasion was documented upon image acquisition with an AxioCam ICm 1 camera coupled to an Axiovert 100 microscope and processed with the AxioVision LE64 program (Carl Zeiss, Oberkochen, Germany). The distance invaded by all of the 50 cells furthest from the spheroid center was measured using ImageJ software [67]. Median invaded distance was computed upon radius subtraction of a reference time point.

### Animal studies

The animal procedures conducted in this study were approved by the Swiss cantonal veterinary office (license numbers ZH178/2016 and ZH109/2020). Crl:CD1-*Foxn1*^*nu*^ mice were purchased from Charles River Laboratories (Sulzfeld, Germany) and Rj:NMRI-*Foxn1*^*nu*^ mice were purchased from Janvier Labs (Le Genest-Saint-Isle, France). A total of 7 (S-24, T-325, ZH-305) or 10 (ZH-161) mice of 4–16 weeks of age were implanted with 200,000 S-24 or T-325 cells, 100,000 ZH-161 cells, or 400,000 ZH-305 cells into the right mouse striatum (3 mm depth 2 mm lateral and 1 mm posterior to the bregma) by means of stereotactic surgery using a 26 s gauge syringe (Hamilton, Reno, NV). The sample was determined based on previous experience in our laboratory when comparing different sublines with targeted modifications in exploratory studies. Investigators were not blinded for the animal studies. Each experimental group included mice implanted with either of the two sublines with the same genotype (i.e., naïve and CRISPR control cells, two CD95 knockout clones, or two CD95L knockout clones). End-stage survival was defined by the onset of neurological symptoms. The onset of diseases other than a brain tumor was pre-established as an exclusion criterion for the assessment of the survival endpoint.

### Statistical analysis

Quantitative results are expressed as mean and standard deviation (SD) or standard error of the mean (SEM). Unless otherwise specified, representative experiments of at least two independent experiments are depicted. Statistical analyses were performed with GraphPad Prism software, version 8 (GraphPad Software, San Diego, CA, www.graphpad.com). Statistical significances were calculated by means of one- or two-way ANOVA with Bonferroni post hoc tests. Statistical significance in in vivo experiments was evaluated by a log-rank test.

## Supplementary information

Author contribution form

Supplementary Figure and Table legends

Figure S1

Figure S2

Figure S3

Figure S4

Figure S5

Figure S6

Figure S7

Figure S8

Figure S9

Table S1

Supplementary Material and Methods

Supplementary Notes

## Data Availability

The datasets used in the analysis of glioma patient overall survival based on *CD95* or *CD95L* mRNA expression levels are available in the TCGA database (https://www.cancer.gov/tcga). All data generated in this manuscript are available for further analysis upon a reasonable request.
