# Supplementary material for: CD95 gene deletion may reduce clonogenic growth and invasiveness of human glioblastoma cells in a CD95 ligand-independent manner
Source: Cell Death Discov. 2022 Jul 29;8:341. doi: 10.1038/s41420-022-01133-y (PMC9338300; doi:10.1038/s41420-022-01133-y)
Supplement: Supplementary file 1 — Author contribution form [file 41420_2022_1133_MOESM1_ESM.pdf]

DECLARATION OF CONTRIBUTIONS TO ARTICLE

ADMC

Manuscript Number:	Journal Name:	
CDD-22-0467	*Cell Death & Differentiation*	(the 'Journal')

Proposed Title of the Contribution:

Detailed Attribution of Authorship (the 'Contribution)

Author(s):

Clara Quijano-Rubio, Manuela Silginer and Michael Weller (the 'Authors')

For all *CDD* articles, each person named as an author in the published version must be able to show he or she has contributed substantially to the article.

Authorship credit should be based on 1) substantial contributions to conception and design, acquisition of data, or analysis and interpretation of data; 2) drafting the article or revising it critically for important intellectual content; and 3) final approval of the version to be published. Authors should meet conditions 1, 2 and 3.

Any person who cannot be shown to have made a substantial contribution to the article cannot be listed as an author in the final version. The name of any person who is deemed to have made a minor contribution can, however, appear in the Acknowledgments section of the article.

Please complete the table below to indicate the contributions of all named authors to the manuscript.

Author Full Name:	Specification of Contribution to the Manuscript:
Clara Quijano-Rubio	Developed the experimental design, performed most of the [illegible]
Manuela Silginer	Coordinated the animal studies and conducted the experiment [illegible]
Michael Weller	Developed the experimental design, analyzed and interpreted the [illegible]

Please complete the table below to indicate the contributions of all named authors to the figures.

Figure 1:

C.Q.R. developed the experimental design, generated, analyzed, assembled and interpreted all the data.
M.W. developed the experimental design, analyzed and interpreted the data.

Figure 2:

C.Q.R. developed the experimental design, generated, analyzed, assembled and interpreted all the data.
M.W. developed the experimental design, analyzed and interpreted the data.

Figure 3:

C.Q.R. developed the experimental design, generated, analyzed, assembled and interpreted all the data.
M.W. developed the experimental design, analyzed and interpreted the data.

Figure 4:

C.Q.R. developed the experimental design, generated, analyzed, assembled and interpreted all the data.
M.W. developed the experimental design, analyzed and interpreted the data.

Figure 5:

C.Q.R. generated the data in A and developed the experimental design, analyzed, assembled and interpreted the data in A and B.
M.S.: coordinated the experiments depicted in A and B and conducted the experiment depicted in B.
M.W. developed the experimental design, analyzed and interpreted the data.

Figure 6:

Signed for and on behalf of the Author(s):	Print Name:	Date:
Clara Quijano-Rubio, Manuela Silginer and Michael Weller	Michael Weller	23-March-2022
