# Supplementary material for: CD95 gene deletion may reduce clonogenic growth and invasiveness of human glioblastoma cells in a CD95 ligand-independent manner
Source: Cell Death Discov. 2022 Jul 29;8:341. doi: 10.1038/s41420-022-01133-y (PMC9338300; doi:10.1038/s41420-022-01133-y)
Supplement: Supplementary file 2 — Supplementary Figure and Table legends [file 41420_2022_1133_MOESM2_ESM.docx]

**Supplementary Figure legends**

Fig. S1. Associations of *CD95* and *CD95L* mRNA expression with overall survival. Kaplan-Meier curves of overall survival stratified by *CD95* or *CD95L* mRNA expression levels in two different cohorts (Glioma - French - 284 - MAS5.0 - u133p2 and Glioblastoma - TCGA - 540 - MAS5.0 - u133a) are depicted. Patients from the Glioma - French - 284 - MAS5.0 - u133p2 cohort were divided into IDH wildtype (glioblastoma) and IDH-mutant glioma based on *IDH1* status selection and additional filtration by histological classification as glioblastoma for the former. Patients from the Glioblastoma - TCGA - 540 - MAS5.0 - u133a cohort were not filtered since *IDH* mutation status data are not available for this cohort. High (red) or low (blue) gene expression was defined using median mRNA expression level of *CD95* (**A**) or *CD95L* (**B**) or the expression level resulting in the highest association with survival (**C**; *CD95,* **D**; *CD95L*) as cut-off. Statistical significances (p) were determined using the log-rank test.

**Fig. S2.** **CD95 and CD95L expression in human glioblastoma cells in vitro.** **A, B** Flow cytometry histograms of CD95 and CD95L cell surface protein levels in S-24, ZH-161, ZH-305 and T-325 cells (corresponding to the data in Figure 1C,D) are depicted. Specific fluorescence indexes (SFI) were calculated by dividing the median fluorescence intensities of the experimental antibody (black) and the isotype control (grey). **C** *CD95L* mRNA expression was assessed by RT-qPCR, using primers annealing to the last two exons of *CD95L*, in a panel of long-term human glioma cell lines (LN-18, LN‑428, D247MG, LN-319, A172, T98G, LN‑229, LN-308) and GIC (S-24, ZH-161, ZH-305, T-269, T-325). *ARF1* was used as internal control. Phorbol myristate acetate (10 ng/ml) and ionomycin (500 ng/ml)-activated peripheral blood mononuclear cells (PBMC) were used as positive control for CD95L expression. RT-qPCR data are expressed as mean and SD (a.t., C_T_ values above reliability threshold, >32).

Fig. S3. CRISPR-Cas9-mediated CD95 and CD95L knockout strategy. Two single guide RNA (sgRNA) sequences with complementarity to a genomic DNA target sequence in the *CD95* or *CD95L gene*, situated immediately next to a protospacer adjacent motif (PAM), were designed and individually cloned into *pSpCas9*(BB)-2A-*GFP* (PX458) plasmids. Both *pSpCas9*(BB)-2A-*GFP-*sgRNA plasmids were transfected into glioma cells. GFP+ cells were selected by fluorescence-activated cell sorting and seeded as single cells to generate CD95 or CD95L knockout clonal populations. DSB double-strand DNA break. Illustration created with BioRender.com.

**Fig. S4. Cell surface CD95 level assessment upon CD95 knockout (KO) in human GIC – additional data.** Flow cytometry histograms corresponding to the specific fluorescence index (SFI) values shown in Figure 3A; right y axis. SFI were calculated dividing the median fluorescence intensities of the experimental antibody (black) and the isotype control (grey). Bulk KO cells, from which CD95 KO clonal sublines were derived, are depicted as a reference for negative SFI values upon staining with the anti-human CD95 clone DX2 relative to a matching isotype control.

Fig. S5. Cell doubling times of CD95 CRISPR knockout (KO) human GIC. Doubling times of CRISPR control or CD95 KO S-24 or T-325 cells were assessed by manual counting and trypan blue exclusion.

Fig. S6. Human GIC sphere formation and invasion pattern. **A** Representative pictures of CRISPR control and CD95 knockout S-24, ZH-161, ZH-305 and T-325 spheres cultured for >10 days in 96-well plates in limiting dilution assays are shown. **B** Images of invading CRISPR control and CD95 KO S-24 or ZH-161 spheroids corresponding to Figure 4C are depicted. **C** Images of non‑invasive ZH-305 or T-325 spheroid-like structures were likewise recorded. Scale bar = 500 μm.

Fig. S7. Gene expression profile of stemness and differentiation markers in human GIC upon *CD95* gene deletion (knockout, KO). mRNA expression of *CD133*, *CD44*, *SOX2*, *MUSHASHI1* and *OCT4* (stemness markers) and *OLIG2*, *NF1*, *TUBB3* and *GFAP* (differentiation markers) was assessed in naïve (non-transfected), CRISPR control and CD95 KO S-24, ZH-305 and T-325 cells by RT-qPCR using *ARF1* as internal control. Data are expressed as mean and SD. a.t., C_T_ values above reliability threshold (C_T_>32).

Fig. S8. Effect of exogenous CD95L stimulation and CD95 overexpression on human GIC growth in vitro. **A** Naïve (non-transfected), CRISPR control or CD95 knockout (KO) S-24 cells were treated with increasing concentrations of soluble CD95L hexamers (Mega-Fas-Ligand, MFL). **B** Naïve or CRISPR control S‑24 cells were transfected with a CD95-expressing pBCMGS plasmid. End‑point metabolic activity was measured by MTT assay in limiting dilution assays. Data are expressed as mean and SEM of representative experiments.

Fig. S9. Effects of CD95 and CD95L knockout (KO) in ZH-305 and T-325 GIC xenograft models**.** Crl:CD1‑*Foxn1^nu^* mice were intracranially implanted with control (naïve or CRISPR control cells), CD95 KO (two different CD95 KO clones) or CD95L KO (two different CD95L KO clones) ZH‑305 or T-325 cells. The survival of n = 7 mice per group was recorded. Median survival (days) is depicted in brackets. UD undefined, indicates the impossibility of computing median survival due to survival exceeding 50% at the latest time point.

**Supplementary Table legend**

**Table S1. List of primers used in RT-qPCR analyses.** The *CD95* primers, which span the predicted Cas9 double-strand DNA break (DSB) sites guided by both *CD95* single guide RNA (sgRNA), were additionally used to verify *CD95* deletion upon CRISPR‑Cas9-mediated knockout. The *CD95L* exon 1_1 and *CD95L* exon 1_2 primers, which overlap the predicted Cas9 DSB sites guided by *CD95L* sgRNA 1 and sgRNA 2, respectively, were additionally used for gene deletion verification upon CRISPR‑Cas9-mediated knockout. All primers were used at a concentration of 100 nM.
