# Supplementary figures and images for: CD95 gene deletion may reduce clonogenic growth and invasiveness of human glioblastoma cells in a CD95 ligand-independent manner
Source: Cell Death Discov. 2022 Jul 29;8:341. doi: 10.1038/s41420-022-01133-y (PMC9338300; doi:10.1038/s41420-022-01133-y)

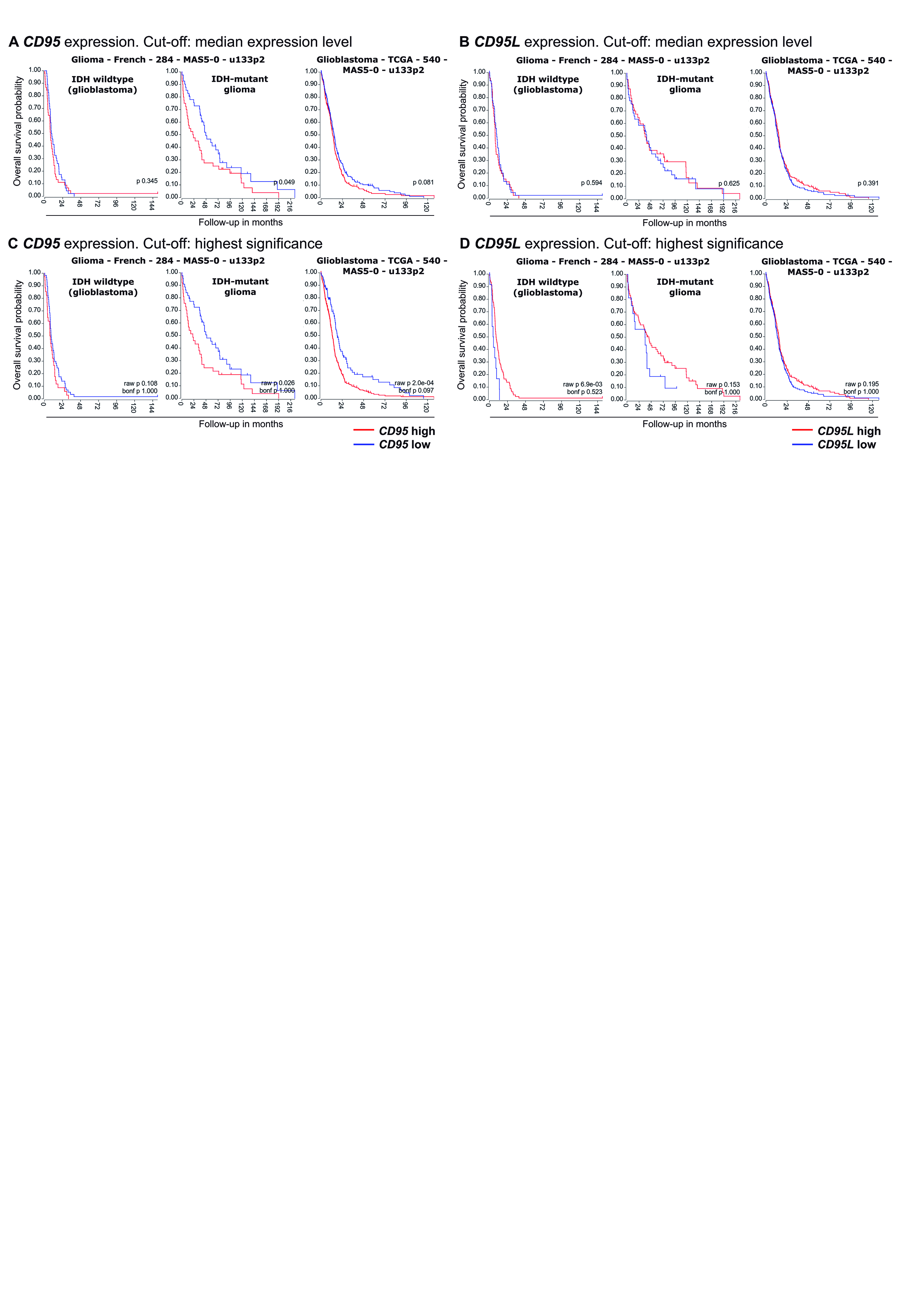

Supplement: Supplementary file 3 — Figure S1 [file 41420_2022_1133_MOESM3_ESM.tif]

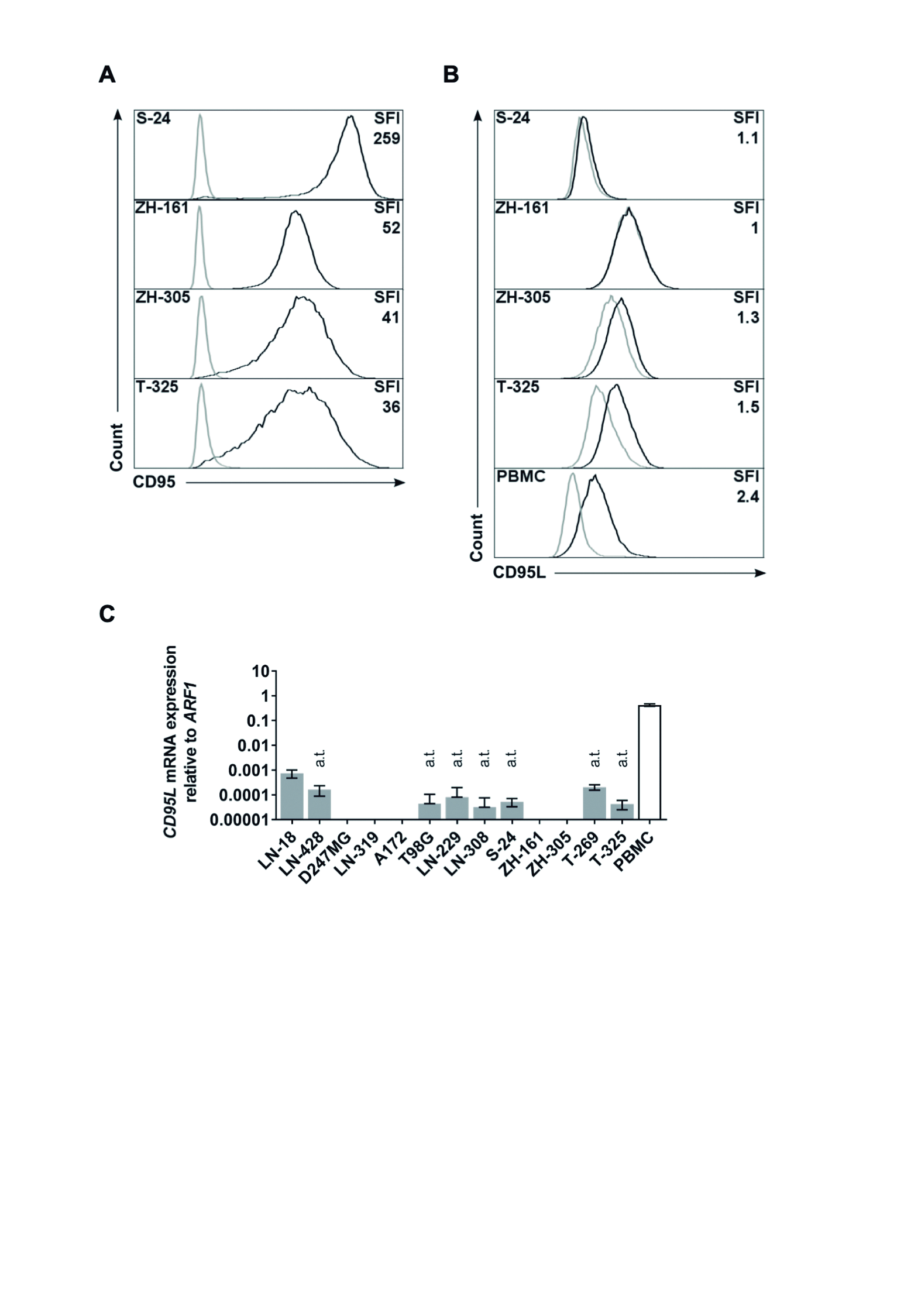

Supplement: Supplementary file 4 — Figure S2 [file 41420_2022_1133_MOESM4_ESM.tif]

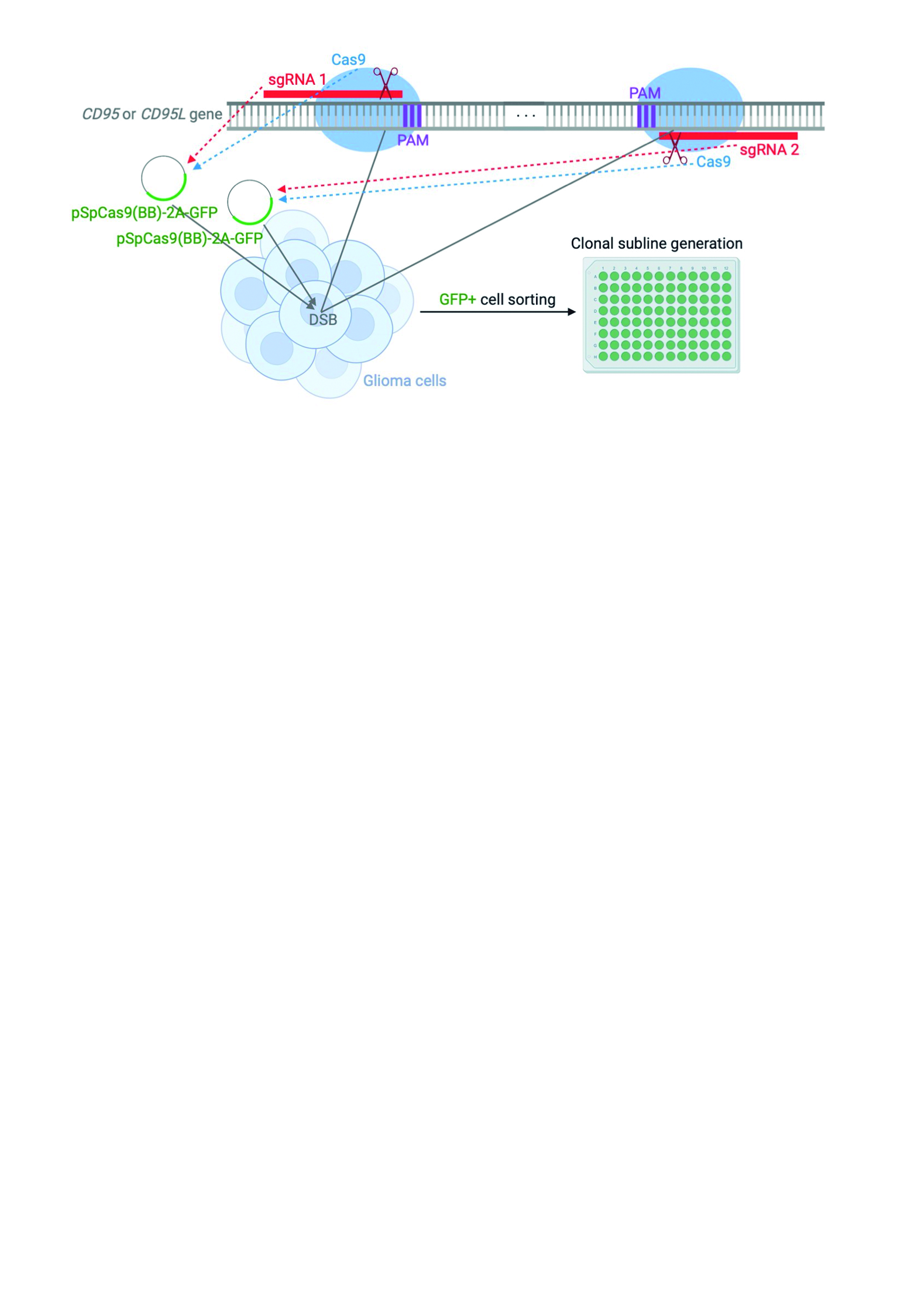

Supplement: Supplementary file 5 — Figure S3 [file 41420_2022_1133_MOESM5_ESM.tif]

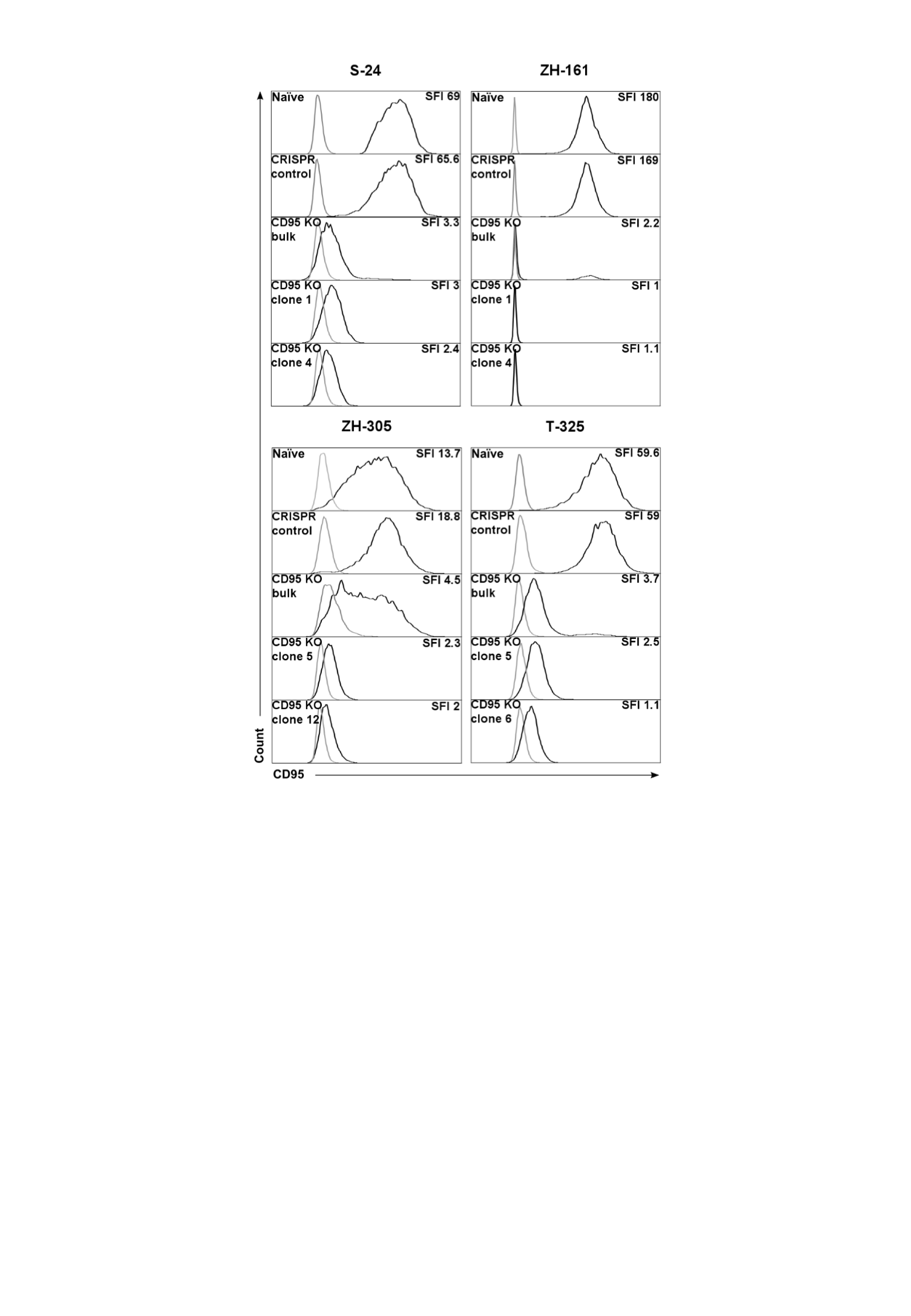

Supplement: Supplementary file 6 — Figure S4 [file 41420_2022_1133_MOESM6_ESM.tiff]

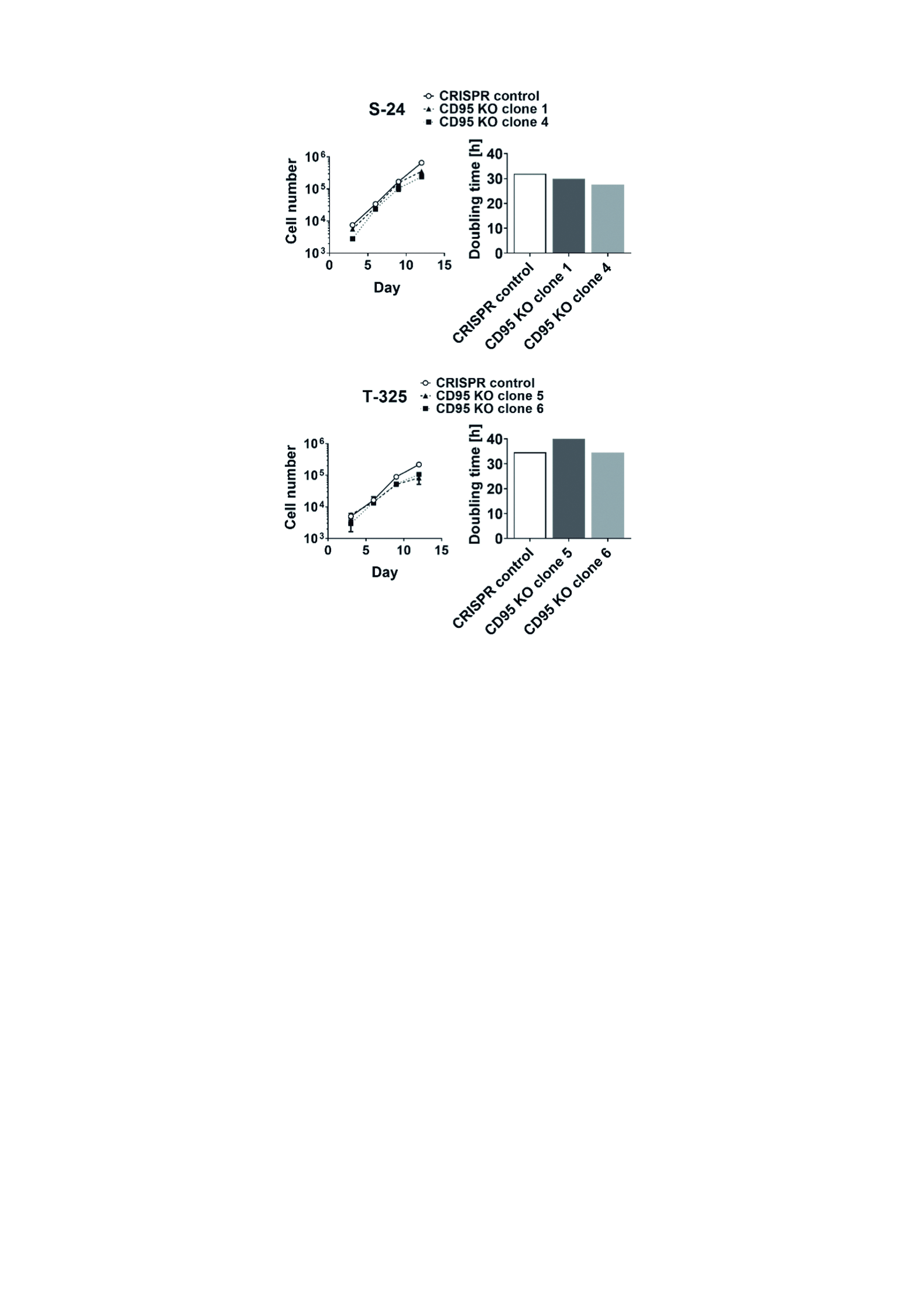

Supplement: Supplementary file 7 — Figure S5 [file 41420_2022_1133_MOESM7_ESM.tif]

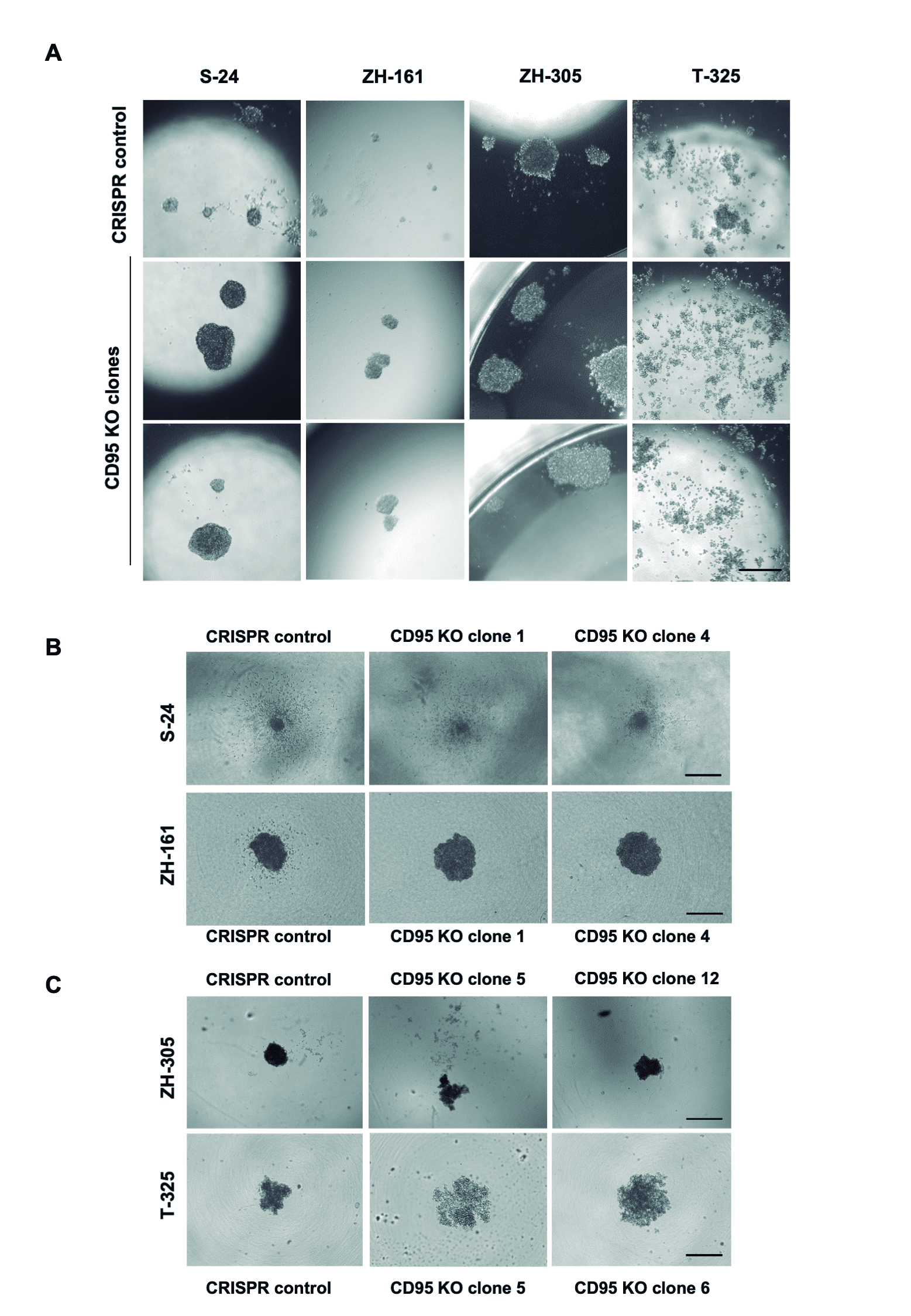

Supplement: Supplementary file 8 — Figure S6 [file 41420_2022_1133_MOESM8_ESM.tif]

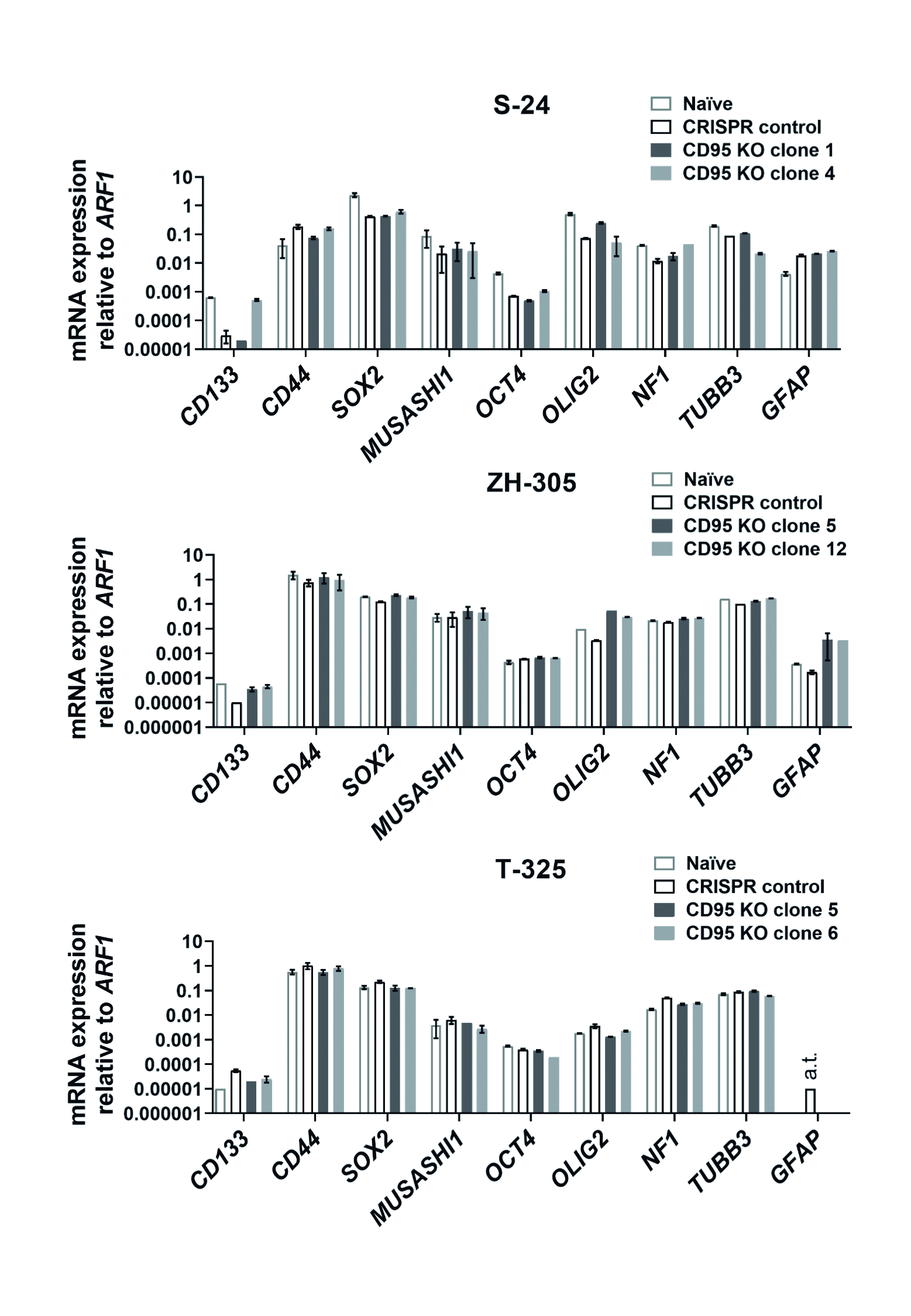

Supplement: Supplementary file 9 — Figure S7 [file 41420_2022_1133_MOESM9_ESM.tif]

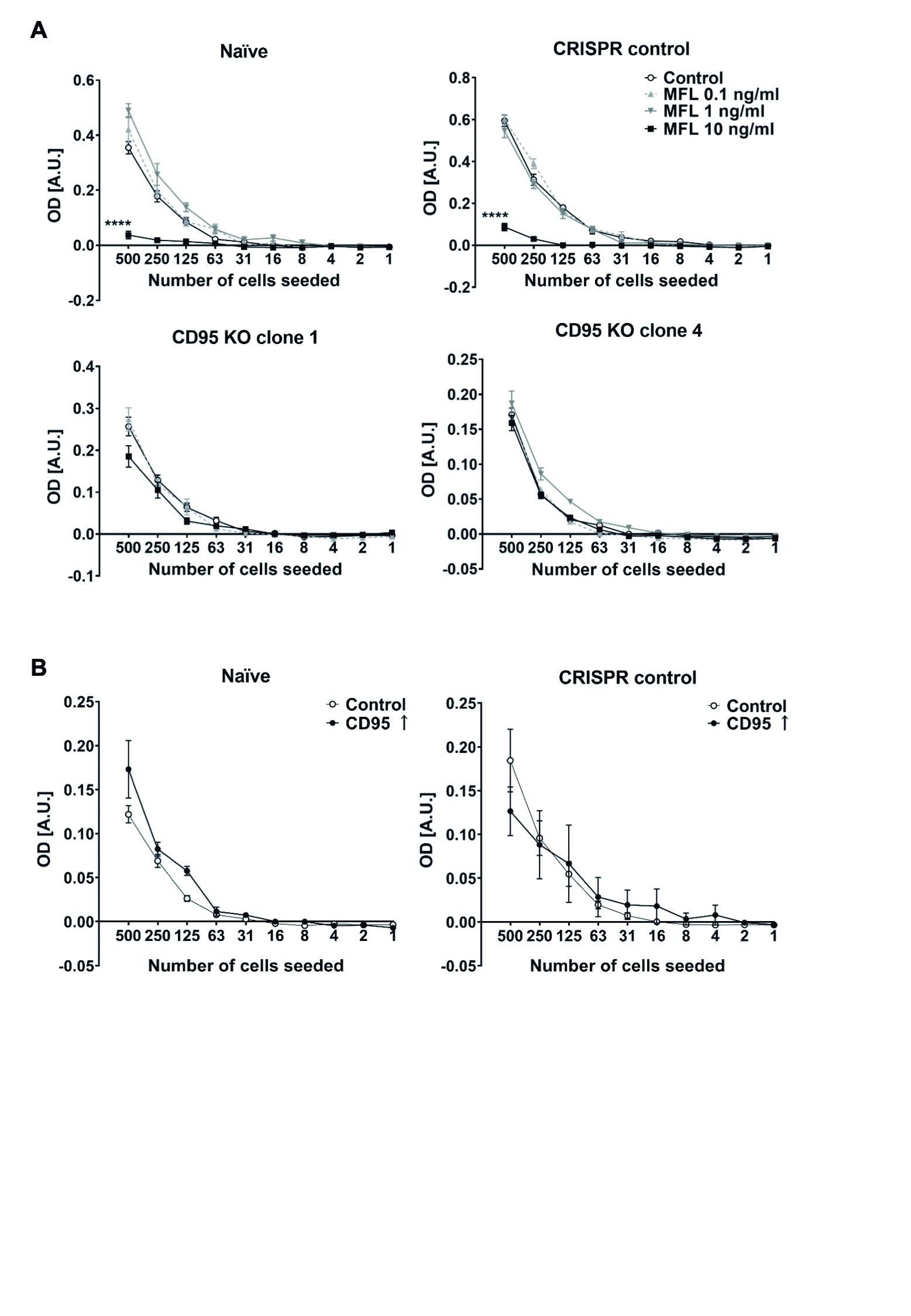

Supplement: Supplementary file 10 — Figure S8 [file 41420_2022_1133_MOESM10_ESM.tif]

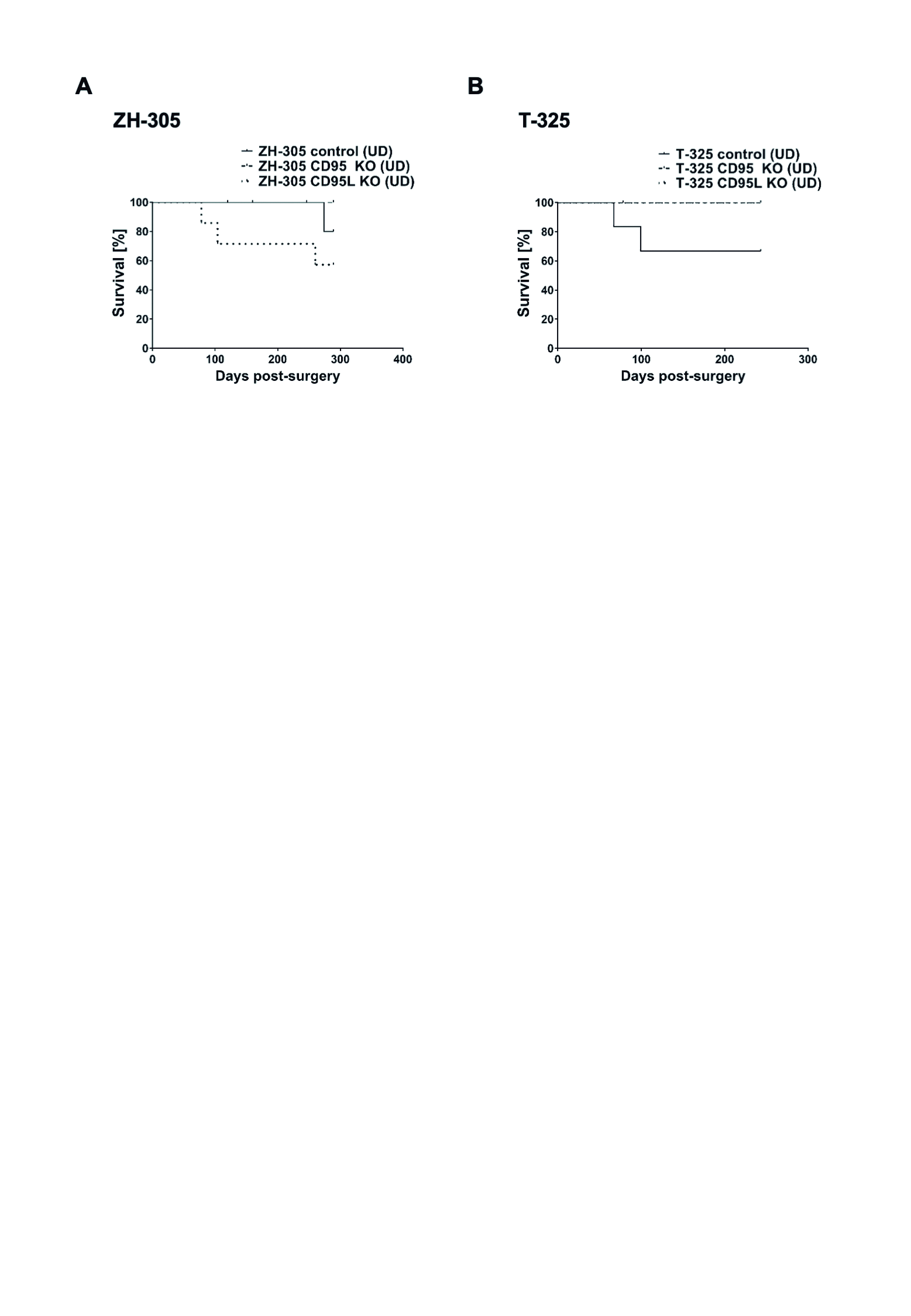

Supplement: Supplementary file 11 — Figure S9 [file 41420_2022_1133_MOESM11_ESM.tif]
