# Supplementary material for: CD95 gene deletion may reduce clonogenic growth and invasiveness of human glioblastoma cells in a CD95 ligand-independent manner
Source: Cell Death Discov. 2022 Jul 29;8:341. doi: 10.1038/s41420-022-01133-y (PMC9338300; doi:10.1038/s41420-022-01133-y)
Supplement: Supplementary file 13 — Supplementary Material and Methods [file 41420_2022_1133_MOESM13_ESM.docx]

**Supplementary Material and Methods**

***CD95* overexpression**

*CD95* gene transfer was carried out by electroporation of the pBCMGS APO-1 sense (cDNA) plasmid or the respective empty control plasmid into naïve (non‑transfected) and CRISPR control S-24 GIC [23]. Electroporation conditions were the same as for CRISPR-Cas9-mediated gene deletion. Positively transfected cells were selected with 750 μg/ml G418 (InvivoGen, San Diego, CA) during four weeks. *CD95* expression was assessed by RT-qPCR and flow cytometry.

**Gene deletion verification by Sanger sequencing**

*CD95L* gene deletion upon CRISPR-Cas9 knockout was determined by means of Sanger sequencing. The DNA of individual clones was isolated with the DNeasy Blood & Tissue Kit (Qiagen, Hilden, Germany) and the sgRNA-targeted *CD95L* gene region was amplified by PCR using the following primers: forward 5’-CCAGGAAGGTGAGCATAGCC-3’, reverse 5’-GAAAAGCACTTTGCAAGCCAG-3’ and the 2X PCR Master Mix from Thermo Fisher Scientific. Amplification was done in a Biometra thermocycler set as follows: 95ºC/120 min and 40 cycles at 95ºC/30 sec, 55ºC/30 sec and 72ºC/30 sec. DNA deletion was interrogated by means of agarose gel electrophoresis and verified by Sanger sequencing (Microsynth), with the forward 5’-GATAGAGAAAGAGAAAGACAGAGG-3’ and reverse 5’-AGTTCTGCCAGCTCCTTCTG-3’ primers.

**Apoptosis assays**

Ten thousand cells were seeded in 96-well plates-wells. Twenty-four hours later, cells were treated with Mega‑Fas-Ligand, cycloheximide, staurosporine, or zVAD-fmk for 6 h. Cells were lysed in 25 mM Tris-HCl pH 8, 120 mM NaCl, 5 mM EDTA and 0.5% NP-40 and exposed to 6.25 µM N-acetyl Asp‑Glu‑Val‑Asp‑7‑amino-4-methylcoumarin (Ac-DEVD-amc). Cleaved Ac-DEVD-amc was fluorometrically quantified at 380ex/450em using an Infinite M200 PRO plate reader (Tecan, Maennedorf, Switzerland).

**Fluorescence-activated cell sorting (FACS)**

Single cell suspensions were obtained upon treatment with Accutase (Thermo Fisher Scientific) and filtered through a 35 μm cell strainer (Corning, NY). Bulk and single cell sorting was performed under sterile conditions with a 100 μm or a 130 μm nozzle in a FACSAria III cell sorter equipped with FACSDiva software (BD Biosciences). Sorted cells were collected in culture medium for subsequent cultivation, expansion or subline generation.

**Cell doubling time assessment**

A total of 5,000 human GIC were seeded in Ø60 mm petri dishes, harvested every third day for a period of 21 days and counted in an hemocytometer. Cell viability was determined using 0.4% trypan blue (Sigma-Aldrich). Doubling times were computed as: $\frac{\left( t-t_{0} \right) \times\log_{2}}{log(n-n_{0})}$, where t is culture time, selected within the exponential cell growth phase, and n is the number of viable cells at t.
