# Supplementary material for: CD95 gene deletion may reduce clonogenic growth and invasiveness of human glioblastoma cells in a CD95 ligand-independent manner
Source: Cell Death Discov. 2022 Jul 29;8:341. doi: 10.1038/s41420-022-01133-y (PMC9338300; doi:10.1038/s41420-022-01133-y)
Supplement: Supplementary file 14 — Supplementary Notes [file 41420_2022_1133_MOESM14_ESM.docx]

**Supplementary Notes**

**Note S1**

In the gene expression analyses conducted in this study, cycle threshold (C_T_) values above 32 were consistently observed to lack reproducibility or target specificity, assessed by comparing amplification in samples possessing or lacking primer binding sites. Consequently, to warrant reliable data interpretation, 32 was established as the C_T_ reliability threshold and samples with C_T_ values above this threshold were considered not to express the examined gene. Since other studies have reported low CD95L transcript abundancy as indicative of gene expression [48], for transparency, in the present study all relative expression values, including those corresponding to C_T_ values above the reliability threshold, are depicted although considered negative and designated as such (a.t., above threshold).

**Note S2**

Cells were lysed with RIPA lysis buffer (Millipore, Burlington, MA) in the presence of 100 μg/mL phenylmethylsulfonyl fluoride and phosphatase and protease inhibitors (Sigma-Aldrich). Uniform protein amounts were loaded into 10% acrylamide/bis gels. Proteins were transferred to 0.45 μM-pore nitrocellulose membranes (BioRad, Hercules, CA) which were blocked in 5% skim milk or bovine serum albumin in 0.1% Tween 20-containing Tris-buffered saline buffer. Membranes were incubated with the anti-CD95 clone CH11 (#05-201 Millipore), polyclonal C‑20 (#715 Santa Cruz Biotechnology) and polyclonal #82419 (Abcam, Cambridge, UK) antibodies or with the anti-CD95L polyclonal #15285 (Abcam) and #834 (Santa Cruz Biotechnology) antibodies, followed by incubation with an appropriate HRP-conjugated secondary antibody (goat anti-mouse IgM #12-489 from Merck, Darmstadt, Germany or goat anti-rabbit IgG #7074 from Santa Cruz Biotechnology). Chemiluminescent detection was achieved upon Pierce ECL immunoblot substrate incubation and exposure to x-ray films. Neither CD95 nor CD95L protein levels were reliably detected in PBMC or glioma cells (data not shown).

**Note S3**

*CD95* gene deletion was directed by two single guide RNA (sgRNA) targeting the start codon in the first *CD95* exon and a downstream sequence situated in the second *CD95* exon, encoding part of the extracellular domain of all known CD95 isoforms. *CD95L* gene deletion was achieved by disrupting the cytoplasmic and transmembrane CD95L domains of all known isoforms upon targeting two gene sequences situated in the first *CD95L* exon.

The two sgRNA were designed to target coding sequences present in all target gene transcript variants. For *CD95* deletion, the sgRNA pair targeted two initial gene regions encoding part of the extracellular CD95 domain. For *CD95L* deletion, the sgRNA pair targeted two initial gene sequences encoding the cytoplasmic and transmembrane CD95L domains.

**Note S4**

The absence of CD95L expression in human glioma cells in vitro rendered the evaluation of the effect of CD95L depletion in human GIC in vitro obsolete. Yet, experiments corresponding to those shown in Fig. 4 for CD95 knockout cells were also conducted in CD95L knockout cells and expectedly revealed no phenotype. Furthermore, exposing CD95‑expressing S-24 cells to sublethal doses of exogenous CD95L did not stimulate clonogenic growth at all and thus not more than in CD95 knockout cells, consistent with the hypothesis that the phenotype of the CD95 knockout is CD95L-independent (Fig. S8A). Additionally, CD95 overexpression did not augment clonogenic growth, suggesting that CD95 signaling may maintain constitutive GIC growth but does not promote it beyond constitutive levels if enforced by overexpression (Fig. S8B). Neither *CD95* re‑transfection in CD95 knockout cells nor *CD95L* transfection in any S-24 GIC subline (Note S5) was tolerated (data not shown).

**Note S5**

*CD95L* transfection was performed by lentivirus-mediated gene delivery. The *CD95L* sequence was obtained from a pLenti-GIII-CMV.RFP-2A-Puro-CD95L plasmid, kindly provided by Marcus E. Peter (Chicago, U.S.A), and inserted in the lentiviral transfer vector #39481 (Addgene) by PCR-based cloning. CaPO_4_ precipitation-mediated transfection of HEK 293T cells with the pMD2.G (#12259, Addgene) and the pCMV-dR8.91 (#2221, Addgene) plasmids was performed for lentivirus generation. Glioma cell transduction was carried out using 8 μg/ml polybrene (Santa Cruz Biotechnology, Dallas, TX). Puromycin (6 μg/ml, Thermo Fisher Scientific) was used for selection.
